# The Effect of MRI Exposure on the Shear Bond Strength and Adhesive Remnant Index of Different Bracket Types

**DOI:** 10.3390/dj13030108

**Published:** 2025-02-28

**Authors:** Luka Šimunović, Jakov Stojanović, Katarina Tečić, Dijana Zadravec, Senka Meštrović

**Affiliations:** 1 Department of Orthodontics, School of Dental Medicine, University of Zagreb, 10000 Zagreb, Croatia; lsimunovic@sfzg.unizg.hr; 2 School of Dental Medicine, University of Zagreb, 10000 Zagreb, Croatia; jstojanovic@sfzg.hr (J.S.); ktecic@sfzg.hr (K.T.); 3 Department of Diagnostic and Interventional Radiology, School of Dental Medicine, University of Zagreb, 10000 Zagreb, Croatia; zadravec@sfzg.hr; 4 University Hospital Center “Sestre Milosrdnice”, School of Dental Medicine, University of Zagreb, 10000 Zagreb, Croatia

**Keywords:** shear bond strength, adhesive remnant index, orthodontic brackets, magnetic resonance imaging

## Abstract

**Background/Objectives**: Magnetic resonance imaging (MRI) is widely used in diagnostics, but its effects on orthodontic materials remain a concern. This study aimed to evaluate the impact of MRI exposure at 1.5 T and 3 T on the shear bond strength (SBS) and adhesive remnant index (ARI) of different orthodontic bracket types (metal, self-ligating, and ceramic). **Methods**: A total of 90 extracted human premolars were divided into three groups (control, 1.5 T, and 3 T MRI exposure). The three bracket types were bonded using Transbond XT adhesive and subjected to standardized polymerization. MRI scans were conducted using 1.5 T and 3 T machines with clinically relevant sequences. SBS was measured using a universal testing machine, and the ARI was assessed under a stereomicroscope. Statistical analysis was performed using Kruskal–Wallis and chi-square tests. **Results**: MRI exposure influenced SBS and the ARI differently across bracket types. Firstly, 3 T MRI exposure significantly reduced SBS in self-ligating (*p* = 0.017) and ceramic brackets (*p* = 0.014) compared to the control, whereas metal brackets showed no significant changes. ARI scores varied across MRI conditions, with metal and self-ligating brackets showing increased adhesive retention at higher field strengths. No significant differences were observed in ARI scores for ceramic brackets across MRI conditions. **Conclusions**: The clinical importance of understanding these results is that both patients and clinicians must be aware of inevitable changes that occur in SBS during MRI, since exposure to high-field MRI, particularly 3 T, may alter bond strength and adhesive failure characteristics.

## 1. Introduction

The assessment of shear bond strength (SBS) critically influences the clinical performance and longevity of orthodontic brackets. At the same time, the adhesive remnant index (ARI) is essential in orthodontics, because of possible iatrogenic incidences during debonding procedures [1,2,3]. SBS quantifies the maximum force an adhesive can withstand before failure, directly measuring the bonding efficacy between brackets and tooth surfaces [4,5]. Meanwhile, the ARI evaluates the amount of adhesive remaining on the tooth after debonding, using a standardized four-point scale to provide insight into the adhesive’s performance and failure mode [6]. High SBS values are desirable to prevent bracket detachment during orthodontic treatment, while ARI scores inform post-debonding adhesive removal and enamel preservation strategies [7,8].

Orthodontic bracket materials significantly impact bonding outcomes and patient satisfaction [9]. Stainless steel brackets, valued for their strength and corrosion resistance, are preferred for cases requiring substantial tooth movement [10]. Conversely, ceramic brackets offer superior aesthetics, closely matching natural tooth color, though their brittleness necessitates cautious application in cases with lower occlusal forces [11]. Self-ligating brackets, with built-in mechanisms to reduce friction, are increasingly popular due to their efficiency and enhanced oral hygiene benefits [12]. The choice of bracket type is influenced by clinical goals, aesthetic preferences, and patient-specific factors.

Magnetic resonance imaging (MRI) has become indispensable in modern diagnostics, raising questions about its effects on orthodontic materials. During MRI exposure, orthodontic brackets, wires, and adhesives may experience physical changes, such as thermal effects or bond weakening [13]. Systematic reviews indicate that while temperature increases in stainless steel and nickel-titanium brackets are statistically significant, they are clinically negligible. However, exposure to higher magnetic field strengths, such as 3 T, has been associated with increased micro-leakage and reduced SBS at the adhesive–enamel and bracket–adhesive interfaces. Such findings suggest that MRI at higher field strengths may compromise the integrity of orthodontic bonding [14,15,16].

Research examining the effects of MRI on orthodontic materials has highlighted critical gaps. While physical changes such as thermal effects are unlikely to influence clinical outcomes significantly, micro-leakage at the adhesive interface poses a potential risk. A comparative study found that 3 T MRI exposure resulted in significantly higher gingival margin micro-leakage compared to 1.5 T exposure. These findings underscore the importance of considering MRI field strength in clinical decision-making, particularly for patients undergoing orthodontic treatment [14].

The use of bovine teeth in in vitro studies evaluating SBS and the ARI introduces additional variables. Studies have demonstrated that bovine enamel bond strength is weaker than human enamel, potentially limiting the generalizability of findings. Structural differences, such as crystal size and elemental composition, further differentiate bovine and human teeth. Despite these differences, bovine teeth remain a viable substitute for human teeth in many studies, provided their limitations are acknowledged and standardized testing protocols are employed [17].

This study aims to evaluate the SBS and ARI of orthodontic brackets subjected to MRI exposure at different magnetic field strengths (1.5 T and 3 T), so the null hypothesis was that exposure to 1.5 T and 3 T MRI does not significantly affect the shear bond strength (SBS) or adhesive remnant index (ARI) of orthodontic brackets (metal, self-ligating, and ceramic) compared to the control group. By addressing the effects of varying MRI field strengths on bonding performance, the findings aim to provide evidence-based guidelines for clinical practice, enhancing patient safety and treatment outcomes. The study builds upon existing knowledge to bridge the gap in understanding the interplay between MRI diagnostics and orthodontic material performance, contributing to the optimization of orthodontic care in contemporary clinical settings.

## 2. Materials and Methods

### 2.1. Specimen Preparation

In this study, we used a sample of 90 healthy permanent human premolars (first and second, upper and lower) extracted primarily for orthodontic reasons. The teeth were collected, rinsed with water, cleaned with fluoride-free paste using a brush, and stored in a saline solution for no longer than three months. Inclusion criteria comprised premolars without caries, white spots, fillings, or other restorations. The teeth were randomly divided into nine groups (*n* = 10), which were exposed to different magnetic resonance imaging (MRI) conditions or served as a control group. The study was conducted in accordance with the Declaration of Helsinki and approved by the Institutional Review Board (or Ethics Committee) of the School of Dental Medicine, University of Zagreb (05-PA-1-4/25, 1 January 2024).

### 2.2. Bonding Procedure

Before bonding the brackets, the teeth were etched with 37% orthophosphoric acid (Etching Gel, Ivoclar Vivadent, Schaan, Liechtenstein) for 15 s, rinsed with water for 20 s, and air-dried for 10 s. Three types of orthodontic brackets from the GCO line were bonded to the teeth: GCO Chic Roth (ceramic), GCO Axcess Roth (metal), and GCO Experience Metal Roth (self-ligating) (GC Orthodontics, Alsip, IL, USA). The brackets were bonded using Transbond XT primer and Transbond XT adhesive (3M Unitek, Monrovia, CA, USA) following the manufacturer’s instructions. The excess adhesive was removed with a probe before polymerization. Polymerization was performed with a curing device with a power of 1000 mW/cm^2^ (Bluephase Style LED, Ivoclar Vivadent, Schaan, Liechtenstein) for 20 s from two directions for metallic brackets and through the ceramic brackets. The curing device’s power was verified using the Bluephase Meter II (Ivoclar Vivadent, Schaan, Liechtenstein) before each polymerization procedure.

### 2.3. Tooth Mounting

The teeth were then embedded in cylindrical molds using cold-curing resin (Orthocryl, Dentaurum GmbH & Co. KG, Ispringen, Germany), ensuring that the buccal surfaces of the teeth were parallel to the base using a mounting guide. After resin curing, the teeth were stored in a saline solution until exposure to MRI.

### 2.4. Magnetic Resonance Imaging (MRI)

MRI exposure was performed using MAGNETOM Aera1.5 Tesla (Siemens Healthineers, Erlangen, Njemačka) and uMR Omega 3 Tesla (United Imaging, Houston, TX, USA). A standard head coil was used, with 16 channels on the Aera and 32 channels on the Omega. The scanning time was 25 min for the Aera and 20 min for the Omega. The samples were placed in a plastic box filled with saline solution, maintaining a focal distance that simulates a standard head and neck MRI setup [14]. The brackets were positioned to replicate their natural intraoral orientation, with the buccal surfaces facing outward, as they would in a clinical setting. This ensured that the electromagnetic interactions affecting the brackets and adhesives closely resembled in vivo conditions during MRI exposure. The imaging sequences included DWI, T1, T2, FLAIR, and GRE T*.

### 2.5. Shear Bond Strength (SBS) Test

The bond strength of the orthodontic brackets was measured using a universal testing machine (Instron Universal Testing Machine, Instron, Norwood, MA, USA) by applying occlusogingival load to the bracket at a constant speed of 1 mm/min until bond failure occurred. Shear bond strength (SBS) was calculated by dividing the bond failure force by the bonding area and expressed in MPa. The bonding area of the orthodontic bracket base was as follows: self-ligating 10.71 mm^2^ (3.15 × 3.40), ceramic 12.25 mm^2^ (3.50 × 3.50), and metal 10.295 mm^2^ (2.90 × 3.55).

### 2.6. Adhesive Remnant Index (ARI)

The ARI evaluation was performed using a stereomicroscope (Olympus SZX7, Olympus Corporation, Tokyo, Japan) at 10× magnification to assess the adhesive remnant index. Two independent examiners—a young researcher (PhD candidate) and a full professor (mentor) with extensive experience in orthodontic research—conducted the ARI scoring. To ensure consistency, inter-examiner reliability was assessed using Cohen’s kappa statistic, yielding a kappa value of 0.87, indicating almost perfect agreement. In cases of discrepancy, consensus was reached through discussion before finalizing the scores. The range of the ARI scores was 0 to 3. To be exact, 0 indicates that there is no adhesive remaining on the enamel surface, 1 indicates that less than half of the adhesive is there, 2 shows that half or more of the adhesive is present, and 3 indicates that all the adhesive is present with visible impression of the bracket mesh.

### 2.7. Sample Size Calculation

The sample size for this study was determined using GPower Version 3.1.9.7, (Heinrich-Heine-Universität Düsseldorf, Germany) statistical software (GPower, Heinrich-Heine-Universität, Düsseldorf, Germany). Given the absence of prior studies specifically examining the impact of MRI exposure on orthodontic bracket bond strength, we selected an effect size of 0.5 (Cohen’s d), which represents a moderate effect size. This decision aligns with established statistical conventions, where an effect size of 0.5 is commonly used in biomedical research to detect meaningful differences while balancing statistical power and practical feasibility [18]. With a significance level of 0.05 (α = 5%) and a statistical power of 0.8 (80%), the analysis determined that a minimum of 90 samples (teeth) was required to detect a clinically significant effect. The total sample was divided into nine groups (*n* = 10 per group), with each group subjected to different experimental conditions, including the control, MRI exposure at 1.5 T, and MRI exposure at 3 T for three types of orthodontic brackets.

### 2.8. Statistical Analysis

Data on bond strength and the adhesive remnant index (ARI) were analyzed using SPSS statistical software version 29.0.1.0 (IBM SPSS Statistics, Armonk, NY, USA). The normality of data distribution was assessed with the Kolmogorov–Smirnov or Shapiro–Wilk test. The Kruskal–Wallis test with Dunn’s test for post hoc analysis was used to compare SBS between groups. The ARI was analyzed using the χ^2^ test (chi-square) or Fisher’s exact test, depending on data distribution. Statistical significance was set at *p* < 0.05. The results are presented as median values and interquartile range for continuous variables and as frequencies and percentages for categorical variables.

## 3. Results

### 3.1. Shear Bond Strength (SBS)

Descriptive statistics (median and IQR) of shear bond strength (SBS) among different bracket types (metal, self-ligating, and ceramic) within the control, 1.5 T, and 3 T groups are presented in Table 1. The median SBS values in the control group were 16.50 MPa (IQR: 14.80–17.70) for metal brackets, 16.65 MPa (IQR: 11.35–20.60) for self-ligating brackets, and 13.13 MPa (IQR: 8.70–13.85) for ceramic brackets. The results showed no statistically significant difference in SBS between the bracket types (H(2) = 2.320, *p* = 0.313) in the control group. In the 1.5 T group, the median SBS values were 14.95 MPa (IQR: 12.40–16.75) for metal brackets, 12.65 MPa (IQR: 5.68–20.90) for self-ligating brackets, and 11.75 MPa (IQR: 8.25–14.25) for ceramic brackets, again with no significant differences (H(2) = 1.153, *p* = 0.562). Similarly, in the 3 T group, the median SBS values were 9.08 MPa (IQR: 4.80–17.00) for metal brackets, 5.30 MPa (IQR: 3.85–9.95) for self-ligating brackets, and 4.60 MPa (IQR: 3.15–4.95) for ceramic brackets, with no statistically significant differences observed (H(2) = 5.707, *p* = 0.058).

**Figure 1:**
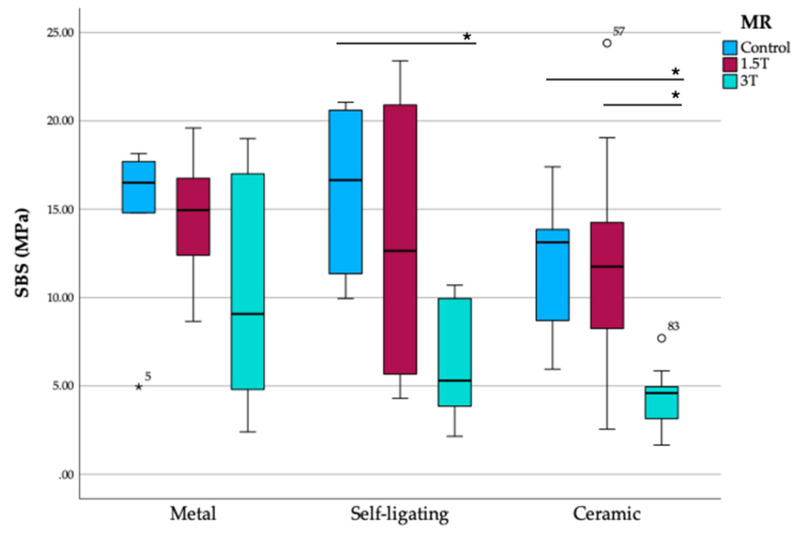
SBS among different bracket types and MRI exposure; bolded * indicates statistically significant data *p* < 0.05.

There was no significant difference in SBS for metal brackets across the conditions. For self-ligating brackets, the 3 T condition significantly reduced SBS compared to the control group (*p* = 0.017), but no difference was observed between 3 T and 1.5 T or between 1.5 T and the control. For ceramic brackets, SBS was significantly lower in the 3 T condition compared to both the control (*p* = 0.014) and 1.5 T (*p* = 0.014) conditions, while no significant difference was observed between the control and 1.5 T groups (Figure 1).

### 3.2. Adhesive Remnant Index (ARI)

The ARI scores were compared among the three bracket groups (metal, self-ligating, and ceramic). For metal brackets, the ARI scores were distributed as follows: 8.0% scored 0, 24.0% scored 1, 16.0% scored 2, 44.0% scored 3, and 8.0% scored 4. For self-ligating brackets, 29.2% scored 0, 16.7% scored 1, 4.2% scored 2, 50.0% scored 3, and none scored 4. For ceramic brackets, 26.9% scored 0, 23.1% scored 1, 11.5% scored 2, 34.6% scored 3, and 3.8% scored 4. Pearson’s chi-square test indicated no statistically significant differences in ARI distribution among the three bracket groups (χ^2^(8) = 7.825, *p* = 0.451).

The ARI distribution for metal brackets showed a significant difference across MRI conditions (χ^2^(8) = 19.245, *p* = 0.014). Pairwise comparisons indicated significant increases in ARI scores of 3 in the 3 T group compared to the control and 1.5 T groups.

The ARI distribution for self-ligating brackets also demonstrated a significant difference across MRI conditions (χ^2^(8) = 15.162, *p* = 0.019). Pairwise comparisons indicated significantly higher scores of 3 in the 1.5 T group compared to the control and 3 T groups.

For ceramic brackets, no significant difference in ARI distribution was observed across MRI conditions (χ^2^(8) = 11.886, *p* = 0.156) (Table 2).

The effect sizes for the ARI distributions were assessed using Cramer’s V. For bracket groups, Cramer’s V was 0.228 (*p* = 0.451), indicating a weak and non-significant association. For MRI conditions, Cramer’s V was 0.353 (*p* = 0.017), indicating a moderate and significant association.

## 4. Discussion

The shear bond strength (SBS) of orthodontic brackets is a critical factor influencing clinical outcomes, and its variability among bracket types has been extensively studied. Metal brackets are often cited for their consistent SBS due to their material properties, such as higher tensile strength and resistance to environmental stressors, while ceramic brackets, despite their aesthetic advantages, are generally associated with lower SBS due to their brittle nature and weaker adhesive bonding to enamel [16,19,20,21]. Self-ligating brackets offer intermediate SBS values, although their bond strength can be influenced by their mechanical configuration and variations in bracket base design [22,23]. Variations in SBS are also attributed to adhesive systems, including resin-based adhesives versus glass ionomer cement, enamel preparation techniques such as acid etching or laser conditioning, and environmental conditions like moisture contamination or thermal cycling [24,25]. The potential for enamel damage during orthodontic bracket debonding is a significant concern, as it is closely associated with SBS and the ARI. High SBS values, while ensuring bracket stability during treatment, may increase the risk of enamel fractures upon removal due to the greater force required for debonding [3]. Conversely, lower SBS values might lead to premature bracket failure but could reduce the likelihood of enamel damage during debonding. The ARI provides insight into the location of bond failure; a higher ARI score indicates more adhesive remaining on the enamel surface, which may necessitate additional cleaning but could protect the enamel from damage. Studies have shown that MRI exposure, particularly at higher field strengths like 3 Tesla (3 T), can influence the adhesion between orthodontic brackets and enamel. For instance, research indicates that 3 T MRI exposure may weaken the bond, potentially altering SBS and ARI values, which could, in turn, affect the integrity of the enamel during bracket removal [26]. A recent review by Fokou et al. [27] has provided insights into quantitative volumetric enamel loss following debonding and clean-up, emphasizing the importance of adhesive selection and removal techniques to minimize iatrogenic enamel damage. Therefore, understanding the impact of MRI exposure on these parameters is crucial for minimizing enamel damage during orthodontic treatment.

The analysis of shear bond strength (SBS) revealed no statistically significant differences among the bracket types (metal, self-ligating, and ceramic) in the control, 1.5 T, or 3 T groups, though trends were apparent. In the control group, metal and self-ligating brackets demonstrated higher median SBS values compared to ceramic brackets, but this difference was not significant (*p* = 0.313). Under 1.5 T conditions, SBS values decreased slightly for all brackets, with no significant differences between groups (*p* = 0.562). In the 3 T group, however, the median SBS values dropped markedly, particularly for self-ligating and ceramic brackets. Ceramic brackets exhibited significantly lower SBS in the 3 T condition compared to both the control and 1.5 T conditions (*p* = 0.014), indicating their susceptibility to high magnetic fields. Self-ligating brackets also showed a significant reduction in SBS under 3 T compared to the control group (*p* = 0.017), though no differences were observed between 3 T and 1.5 T or 1.5 T and the control. Metal brackets maintained relatively stable SBS across all conditions, suggesting greater resilience to magnetic field exposure. These findings underscore the potential influence of MRI conditions, particularly at 3 T, on bond strength, which is more pronounced for ceramic and self-ligating brackets.

In contrast, the ARI provides insight into the failure mode of the adhesive bond, reflecting whether the adhesive failure is cohesive (within the adhesive) or adhesive (at the bracket–adhesive or enamel–adhesive interface). Studies consistently report that metal brackets tend to demonstrate higher ARI scores, indicative of cohesive failure within the adhesive due to their robust bond to enamel, whereas ceramic brackets often show adhesive failure due to their smooth base design and weaker interface bond [28,29,30,31]. Factors such as adhesive type, curing method (e.g., light-cured vs. self-cured), and enamel surface conditioning significantly influence both SBS and the ARI, affecting clinical outcomes like debonding rates and enamel damage [32,33,34,35].

The ARI analysis highlighted distinct adhesive failure patterns among metal, self-ligating, and ceramic brackets, with no statistically significant differences observed among the groups (*p* = 0.451). Metal brackets showed a tendency for higher ARI scores of 3 under 3 T MRI conditions, indicating greater adhesive retention on the bracket, while self-ligating brackets exhibited significantly higher ARI scores of 3 under 1.5 T conditions. Ceramic brackets, in contrast, demonstrated consistent adhesive performance across all MRI conditions, likely due to their non-magnetic properties. The moderate association between MRI conditions and ARI distributions (Cramer’s V = 0.353, *p* = 0.017) underscores the influence of magnetic field strength on adhesive failure, particularly for metal and self-ligating brackets, which appear more susceptible to these effects. Moreover, clinical environments, including exposure to electromagnetic fields like those encountered in MRI, can alter both SBS and ARI distributions. For example, higher magnetic field strengths, such as 3 T, may induce thermal and electromagnetic stress on adhesive materials, leading to the degradation of adhesive properties and bond strength [36,37]. The results of this study align with prior findings, showing that metal brackets exhibit resilience under varying conditions, while self-ligating and ceramic brackets are more susceptible to bond strength reduction, particularly in high-field MRI environments. These effects may be explained by differences in the thermal conductivity and structural stability of bracket materials, as well as potential alterations in adhesive polymerization [38,39]. The observed reduction in SBS for self-ligating and ceramic brackets following 3 T MRI exposure raises concerns regarding bracket survival in vivo and the need for modifications in the bonding protocol to enhance retention in high-field MRI environments. Brackets with weakened bond strength may be more susceptible to spontaneous debonding during orthodontic treatment, potentially leading to treatment delays, increased patient visits, and compromised tooth movement control. Given that some orthodontic patients—such as those undergoing neurological or oncological monitoring—may require multiple MRI scans, clinicians should consider alternative adhesive protocols to mitigate bond degradation. One possible approach is adjusting the curing time, as prolonged light-curing has been shown to increase polymerization efficiency and bond strength, particularly in adhesives with high filler content [40]. However, excessive curing may also lead to greater polymerization shrinkage, which could compromise bond integrity. Alternatively, the use of different primer systems, such as self-etching primers or universal adhesives containing silane coupling agents, may enhance adhesion and improve bond durability against external environmental factors, including electromagnetic exposure from MRI [4,6]. Studies have indicated that primers incorporating nanofillers or bioactive components may provide additional resistance to degradation, which could be beneficial for orthodontic patients requiring repeated MRI scans. The use of resin-modified glass ionomer cements (RMGICs) or nanofiller-enhanced adhesives may also enhance retention in high-MRI exposure conditions [41].

This study has several limitations, including its in vitro design, which may not fully replicate the intraoral environment, limiting direct clinical applicability. Moreover, this in vitro study simulated MRI conditions specifically for head and neck examinations, as the samples were positioned at a focal distance consistent with standard clinical head and neck MRI protocols. However, this setup does not account for all possible MRI examinations performed on orthodontic patients, such as imaging of other body regions where magnetic field interactions may differ. Additionally, the study only assessed the short-term effects of MRI exposure on SBS and the ARI, while long-term effects and repeated MRI exposure remain unexplored. The use of a single adhesive system (Transbond XT) may also limit the generalizability of the findings, as different adhesives exhibit variable polymerization and degradation behaviors. Additionally, the findings suggest the potential for future innovations in adhesive formulations to improve bond durability under diverse environmental conditions [42,43]. Future research should focus on clinical studies assessing SBS and ARI in orthodontic patients before and after MRI exposure, the effects of different bracket materials and adhesives, and the potential impact of multiple MRI scans over time. Advanced methodologies, such as finite element analysis (FEA), micro-CT imaging, and nanofiller-enhanced adhesives, could further enhance our understanding of the interactions between MRI and orthodontic bonding. Further research is needed to confirm these trends and explore additional factors influencing adhesive performance in clinical settings.

## 5. Conclusions

Patients and clinicians should be aware that high-field MRI (particularly 3 T) alters shear bond strength and adhesive failure characteristics, which may impact orthodontic treatment outcomes:

- Exposure to 3 T MRI significantly reduced shear bond strength (SBS) in self-ligating (*p* = 0.017) and ceramic brackets (*p* = 0.014) compared to the control group, while metal brackets exhibited no statistically significant changes across MRI conditions.
- ARI scores varied depending on bracket type and MRI exposure, with metal brackets showing significantly higher adhesive retention (*p* = 0.014) at 3 T MRI, while ceramic brackets did not exhibit significant changes across MRI conditions.
- Among the tested brackets, ceramic brackets had the lowest SBS values overall, with a significant reduction after 3 T exposure, whereas metal brackets demonstrated the highest SBS values and the least impact from MRI exposure.
- The findings suggest that high-field MRI (3 T) has a more pronounced effect on SBS, particularly for self-ligating and ceramic brackets, which may be clinically relevant when selecting bracket types for orthodontic patients who require MRI scans.

## Figures and Tables

**Table 1:** Descriptive statistics (median and IQR) of shear bond strength (SBS) among the different bracket types (metal, self-ligating, and ceramic) within the control, 1.5 T, and 3 T groups.

**Group**	**MRI**	**Median**	**Percentile 25th**	**Percentile 75th**
Metal	Control	16.5	14.8	17.7
	1.5 T	14.95	12.4	16.75
	3 T	9.08	4.8	17
Self-ligating	Control	16.65	11.35	20.6
	1.5 T	12.65	5.68	20.9
	3 T	5.3	3.85	9.95
Ceramic	Control	13.13	8.7	13.85
	1.5 T	11.75	8.25	14.25
	3 T	4.6	3.15	4.95

**Table 2:** Frequency of ARI scores among the different bracket types (metal, self-ligating, and ceramic) within the control, 1.5 T, and 3 T groups.

**Group**	**MRI**	**ARI 0**	**ARI 1**	**ARI 2**	**ARI 3**	**ARI 4**
Metal	Control	33.3%	33.3%	16.7%	16.7%	0.0%
	1.5 T	0.0%	44.4%	11.1%	22.2%	22.2%
	3 T	0.0%	0.0%	20.0%	80.0%	0.0%
Self-ligating	Control	50.0%	16.7%	16.7%	16.7%	0.0%
	1.5 T	0.0%	0.0%	0.0%	100.0%	0.0%
	3 T	40.0%	30.0%	0.0%	30.0%	0.0%
Ceramic	Control	50.0%	50.0%	0.0%	0.0%	0.0%
	1.5 T	10.0%	20.0%	10.0%	60.0%	0.0%
	3 T	30.0%	10.0%	20.0%	30.0%	10.0%

## Data Availability

The raw data supporting the conclusions of this article will be made available by the authors upon request.

## Abbreviations

The following abbreviations are used in this manuscript:

SBS	Shear Bond Strength
ARI	Adhesive Remnant Index
MRI	Magnetic resonance imaging
RMGICs	Resin-modified glass ionomer cements
